# Trait‐based predictions and responses from laboratory mite populations to harvesting in stochastic environments

**DOI:** 10.1111/1365-2656.12802

**Published:** 2018-06-17

**Authors:** Isabel M. Smallegange, Hedwig M. Ens

**Affiliations:** ^1^ Institute for Biodiversity and Ecosystem Dynamics (IBED) University of Amsterdam Amsterdam The Netherlands

**Keywords:** DEB theory, environmental noise, matrix model, population projection model, red noise, von Bertalanffy growth

## Abstract

1. Predictions on population responses to perturbations are often derived from trait‐based approaches like integral projection models (IPMs), but are rarely tested. IPMs are constructed from functions that describe survival, growth and reproduction in relation to the traits of individuals and their environment. Although these functions comprise biologically non‐informative statistical coefficients within standard IPMs, model parameters of the recently developed dynamic energy budget IPM (DEB‐IPM) are life‐history traits like “length at maturation” and “maximum reproduction rate”. Testing predictions from mechanistic IPMs against empirical observations can therefore provide functional insights into the links between individual life history, the environment and population dynamics.
2. Here, we compared the population dynamics of the bulb mite (*Rhizoglyphus robini*) predicted by a DEB‐IPM with those observed in an experiment where populations experienced daily food rations that were either positively correlated over time (red noise), negatively (blue noise) or uncorrelated (white noise). We also selectively harvested large adults in half of these populations. The model failed to generate detailed predictions of population structure as juvenile numbers were overestimated; likely because juvenile–adult interference competition was underestimated. The model performed well at the population level as, for both harvested and unharvested populations, simulations matched the observed, long‐term stochastic growth rate λ_s_.
3. We next generalised the model to investigate how stochastic change affects mite λ_s_, which correlated well with the frequency *f* of experiencing periods of good environment, but, due to the relationship between *f* and noise colour ρ, did not correlate well with shifts in ρ. The sensitivity of λ_s_ to perturbations in life‐history parameters depended on the type of stochastic change, as well as population growth.
4. Our findings show that responses to differential mortality depend on individual life‐history traits, environmental characteristics and population growth. As long‐term climate change causes ever greater environmental fluctuations, trait‐based approaches will be increasingly important in predicting population responses to change. We therefore conclude by illustrating what questions can be examined with mechanistic trait‐based models like the DEB‐IPM, the answers to which will advance our knowledge of the functional links between individual traits, the environment and population dynamics.

## 1 INTRODUCTION

Understanding population responses to perturbations through differential mortality caused by pathogens, parasites and, in an applied context, harvesting is important for accurately predicting population extinction risks (Clements & Ozgul, 2016), predicting epidemic time courses (Keeling & Gilligan, 2000) and predicting sustainable responses to harvesting (Higgins, Hastings, Sarvela, & Botsford, 1997). The impact that such perturbations can have on populations can be critically influenced by fluctuations in environmental conditions (Kaitala, Ylikarjula, Ranta, & Lundberg, 1997), such as the colour of environmental noise or the frequency with which good environmental conditions occur (Cameron & Benton, 2004; Smallegange, Deere, & Coulson, 2014). Indeed, many insights in population and evolutionary dynamics have been obtained from modelling or simulation studies of population responses to perturbations in stochastic environments (e.g. Dalgleish, Koons, & Adler, 2010; Smallegange et al., 2014; Tuljapurkar, Gaillard, & Coulson, 2009). However, to use predictions from such studies successfully to e.g. devise sustainable harvesting strategies or conservation measures, it is necessary that these model predictions are empirically validated, which, however, is rarely done (e.g. Benton, Cameron, & Grant, 2004; Smallegange & Coulson, 2013).

Testable predictions on population responses to perturbations are often created using a trait‐based approach (Cameron & Benton, 2004; Ozgul, Coulson, Reynolds, Cameron, & Benton, 2012; Webb, Hoeting, Ames, Pyne, & Poff, 2010). In such an approach, individuals, or groups of individuals, are characterised by a trait, which biologists generally take to be any detectable phenotypic property of an organism (King, Stansfield, & Mulligan, 2006), such as an individual’s size. This trait information is then used to parameterise a model; the parameters of which are perturbed in turn in order to predict the effects on the population growth rate (Caswell, 2001). For example, Benton et al. (2004) set out to accurately predict the responses of laboratory populations of soil mites (*Sancassania berlesei*) to selective harvesting (Cameron & Benton, 2004) using a population projection model (PPM) structured by life stage. However, they concluded that accurate predictions will require the additional inclusion of environmental stochasticity, as well as the modelling of processes within life stages (Benton et al., 2004). Ozgul et al. (2012) used both a PPM and an Integral Projection Model (IPM) to predict responses of soil mite populations to perturbations under transient dynamics. IPMs, in contrast to PPMs, include both discrete and continuous variables that can describe individual characteristics within and between life stages (Easterling, Ellner, & Dixon, 2000). They found that IPM predictions on population responses to perturbations provided a better fit with empirical observations than PPM predictions (Ozgul et al., 2012).

Integral projection models have emerged as a powerful trait‐based approach (Ellner, Childs, & Rees, 2016). In IPMs, the demographic processes of growth, survival and reproduction are expressed as functions of the trait(s) of individuals (Ellner et al., 2016). One reason that IPMs are popular is that these functions are estimated using flexible and easy‐to‐use phenomenological methods such as regression models (Ellner et al., 2016). However, the downside of these regression models is that the functions are derived phenomenologically from observations under the current environmental conditions and lack a mechanistic representation of the biological processes that give rise to observed demographic variation. For example, in most IPMs, body size is the trait that is used to characterise individuals (Ellner et al., 2016). Environmental effects are often mediated through body size (e.g. through changes in food availability, de Roos & Persson, 2013) and shifts in body size distributions can affect larger scale dynamics (Woodward et al., 2005). When you use standard IPMs to predict ecological and evolutionary change under changing conditions, one automatically assumes that the (phenomenological) demographic functions estimated for the original (constant or stationary) environment apply to individuals in the novel environments under the changing conditions. However, it has been shown that the dynamics of a population’s body size distribution and the dynamics of population abundance are highly intertwined (de Roos & Persson, 2013). In particular, the relationship between individual body size, survival, growth, and reproduction is significantly affected by population feedback through resource exploitation. The assumption of constant demography of standard IPMs therefore likely does not hold under novel conditions. For example, reproduction rates and/or size distributions could differ greatly between the original and novel environments, or they could vary greatly as environmental conditions vary over time. Resulting extrapolation errors could create a substantial mismatch between predicted and actual population responses to environmental change (e.g. Smallegange et al., 2014).

Most recently, the demographic functions describing growth and reproduction have been derived from a dynamic energy budget (DEB) growth model (Smallegange, Caswell, Toorians, & de Roos, 2017). This model follows the principle of energy conservation and comprises a set of rules on energy allocation such that the more (or less) energy is allocated to growth, the less (or more) will be available to allocate to reproduction (Kooijman & Metz, 1984). Its structure is therefore different, which means that, by using the same types of analyses like perturbation analysis, it is now possible to investigate how life‐history traits such as “length at birth” or “maximum reproduction rate” influence population growth under current and novel conditions. In comparison, standard IPMs would only inform on how biologically non‐informative statistical parameters of regression functions (intercept, slope) relating growth and reproduction to body size, affect population growth. Mechanistic trait‐based models like DEB‐IPMs can therefore provide functional insights into the links between individual life history, the environment and population dynamics. For example, knowing which model parameter, and hence life‐history trait, is most influential to population growth would not only inform on whether a population is e.g. stressed if there is a shift in the distribution of a life‐history trait but also on possible mitigation measures targeted at the appropriate life stages (e.g. juveniles if length at birth is most important, or reproducing females if reproduction rate is most important) to halt any further population decline.

Here, we used a DEB‐IPM to test to what extent a mechanistic, individual‐level description of life‐history traits accurately predicts population‐level responses to perturbations in stochastic environments. We used the bulb mite (*Rhizoglyphus robini*, Acaridae) as a model system, and selectively harvested large adults in order to observe the populations’ responses under both constant and temporally varying food conditions that were characterised by either a blue, red or white noise structure over time. These noise structures respectively represent time series with high‐frequency oscillations and negative autocorrelation structure (blue), low‐frequency oscillations and positive autocorrelation structure (red), and an equal mix of frequencies and no autocorrelation (white). We harvested large adults to accurately reflect the common practice of large‐adult harvesting in hunted and commercially harvested populations (Fenberg & Roy, 2008; Shelton & Mangel, 2011). Using the parameterised DEB‐IPM (Smallegange et al., 2017), we simulated the experiment and projected a population forward over the same blue, red and white stochastic time series in order to predict how selectively harvesting large adults affects population structure and growth. We did not compare predictions from the DEB‐IPM with those from a standard IPM as we cannot track the fate of individual mites within populations, which is required to estimate the strength of density dependence in the statistical functions describing survival, growth and reproduction. A DEB‐IPM would therefore always perform better than a density‐independent standard IPM, as it includes density dependence through food exploitation. Also, comparing both types of IPMs would not answer our question of whether a mechanistic, functionally explicit IPM can accurately predict population responses to change. Finally, we used the results of our individual‐to‐population cross‐level test to create a stochastic demographic model to assess how the serial correlation of good and bad environment states (i.e. the noise structure) and the temporal frequency of good environment states affect the long‐term stochastic population growth rate (average fitness), and its sensitivity to individual life‐history parameters, for both unharvested and large‐adult‐harvested populations.

## 2 MATERIALS AND METHODS

### 2.1 Model species

The bulb mite lives in the soil and feeds on bulbs and tubers and is a pest species of many crops and ornamentals. They are small (100–1,000 μm) and live for up to a few months. From egg to adult, they go through a larval and 2–3 nymph stages, which take between 11 and 40 days depending on food quality (Smallegange, 2011).

### 2.2 Experimental set‐up and statistical analysis

We applied a harvesting treatment to single populations: no harvesting vs. harvesting of large adults, as well as an environmental variation treatment: no variation with constant food, and temporal variation in food availability that had a blue, white or red colour. The treatment combinations were replicated three times, resulting in 24 populations. All populations were censused every fourth day. Harvesting was conducted every other census day, i.e. every 8th day (see details below). Following previous studies (Benton et al., 2004; Cameron & Benton, 2004; Cameron, O’Sullivan, Reynolds, Piertney, & Benton, 2013), we applied a constant‐effort harvesting strategy by removing 50% of the largest adult males and females after the census count; actual numbers were rounded to the nearest integer, and adult size was assessed visually. This harvest rate translates to a survival probability of $\sqrt[{8}]{0.50}=0.92$ per day, which is much lower than the background mortality of bulb mites on e.g. a very low‐quality food source of filter paper, where survival probability is on average 0.97 per day (Deere & Smallegange, 2014). To create the different colours of environmental variation, we first generated a time series of white colour by randomly assigning two or eight yeast rods to 37 time‐steps (mean rod length ± *SD* = 1.375 ± 0.135 mm and mean rod width ± *SD* = 0.348 ± 0.044 mm). We considered two rods a day a bad food state environment and eight rods a day a good food state environment. Spectral mimicry (Cohen, Newman, Cohen, Petchey, & Gonzalez, 1999) was used to create the red and blue food time series as permutations of the white series, which ensured that the mean (5.24), minimum (2.00), maximum (8.00) and variance (9.19) of the number of rods fed per day were identical for all environmental variation time series, thereby controlling for non‐spectral environmental variation properties. Each time‐step within the time series generated equalled 4 days, so if, for example, two rods were assigned to a time‐step, then the populations were fed two yeast rods per day for 4 consecutive days. This was performed so that the rate of change in food availability was not too rapid for the bulb mites to respond to in terms of their generation time (about 11 days under *ad lib* yeast conditions), and also not too slow. Therefore, the experiment lasted 37 × 4 = 148 days, and the actual autocorrelations within each noise colour treatment equalled ρ = −0.85 under blue noise, ρ = 0.05 under white noise and ρ = 0.61 under red noise (Figure 2). Because we tested how well our experimental results could be predicted using our model, we kept the food time series within each treatment identical among replicates. Food levels in the control treatment were kept constant at five yeast rods per day, matching the mean number of rods provided daily within each time series. At the start of the acclimation period, when the populations were small, the required number of rods was replenished every day.

Each population was initiated with 5 adult males and 15 adult females from a stock culture (see Smallegange, 2011 for details of the maintenance and origin of the stock cultures). To reduce transient dynamics, the founder adults were removed after 7–9 days (before the next generation had matured) as they are older, typically larger, and thereby competitively superior, over recently emerged mites. Each population was initially fed five rods of yeast per day for a period of 31–34 days, after which the experiment started. A census of each population was conducted every 4th day by counting all eggs, juveniles (larvae, protonymphs and tritonymphs, including moulting stages) and adults using a hand counter at 15× magnification (Meiji EMZ‐8TRD [10–45×] stereomicroscope). Because juvenile males and females do not phenotypically differ, we assigned half of all eggs and juveniles as female, as the sex ratio in bulb mites is genetically determined (Oliver, 1977). Harvesting was conducted after each census, after which the populations were fed the appropriate number of yeast rods. The experimental populations were maintained in 20‐mm diameter, 50‐mm‐high flat‐bottomed glass test tubes containing plaster of Paris and powdered charcoal bases, which were kept moist to maintain humidity and avoid desiccation of the mites. The tops of the tubes were sealed with a ring of fine mesh that allowed gaseous diffusion, which was held in place by the tubes’ plastic caps with ventilation holes cut into them. The experiment was conducted from September 2011 to February 2012.

We analysed the results using a GLMM with Gaussian errors using the *lme4* package in r (R Core Team, 2013). We included harvesting treatment (no harvesting or harvesting) and environmental variation treatment (control, blue, white or red noise) as fixed factors and “measurement day” and “population tube” as random effects to account for the repeated measures within each experimental population of the numbers of eggs, juveniles and adults, and the stochastic population growth rate λ_s_. The three count response variables were log‐transformed before analysis. The log of the stochastic population growth rate λ_s_ was calculated as $\log\left(\lambda_{s}\right)=\frac{1}{\tau }\sum_{\tau =0}^{\tau -1}r_{t}$ with $r_{t}=\log\left[N\left(t+1\right)/N\left(t\right)\right]$, where *N* is the total population size and τ is the length of the experimental period (Tuljapurkar, Horvitz, & Pascarella, 2003). The log of λ_s_ therefore represents the average of all log daily population growth rates within the experimental period. The model assumptions of Gaussian errors and homoscedacity were confirmed by inspecting the probability plots and error structures. Reported *t*‐values in the results section were calculated from the *p*‐values of the GLMM tables using the *pt* function in r. One population tube (red noise; no harvesting) was dropped during a census and excluded from the analyses.

### 2.3 Brief description of the DEB‐IPM

The rationale of the DEB‐IPM is as follows: if a female survives from day *t* to *t *+ 1, she grows from length *L* to length *L*′ following a von Bertalanffy growth curve. If a female is an adult, she also produces eggs. These events are captured by four fundamental functions: the survival function, $S\left(L\left(t\right)\right)$, the growth function, $G\left(L^{\prime },\;L\left(t\right)\right)$, the reproduction function, $R\left(L\left(t\right)\right)$ and the parent–offspring function, $D\left(L^{\prime },\;L\left(t\right)\right)$, which describes the association between the length *L* of the parent and offspring length *L*′. Denoting the number of females at time t by $N\left(L,\;t\right)$ means that the dynamics of this length number distribution from time *t* to *t *+ 1 can be written as:

(1) $$N\left(L^{\prime },\;t+1\right)=\int_{\Omega }\left[D\left(L^{\prime }\;,\;L\left(t\right)\right)R\left(L\left(t\right)\right)+G\left(L^{\prime }\;,\;L\left(t\right)\right)S\left(L\left(t\right)\right)\right]N\left(L,\;t\right)dL$$

where the closed interval Ω denotes the length domain. The survival function $S\left(L\left(t\right)\right)$ in Equation 1 is the probability that an individual of length *L* survives from time *t* to *t* + 1:

(2) $$S\left(L\left(t\right)\right)=\left\{\begin{array}{cc} e^{-\mu } & \mathrm{for}\;L\leq L_{m}E\left(Y\right)/\kappa \\ 0 & \mathrm{otherwise} \end{array}\right.,$$

where μ is a constant background mortality, *E*(*Y*) is the expected feeding level, which ranges from zero (empty gut) to one (full gut), and represents a proxy for the quality of the environment. The parameter *L*_*m*_ is the maximum length under conditions of unlimited resource, *E*(*Y*) = 1, and κ is the fraction of ingested energy allocated to maintenance and growth. Individuals die from starvation at a length at which maintenance requirements exceed the total amount of assimilated energy, which occurs when *L* > *L*_*m*_ · *Y*/κ.

The function $G\left(L^{\prime },\;L\left(t\right)\right)$ is the probability that an individual of length *L* at time *t* grows to length *L*′ at *t* + 1, conditional on survival:

(3) $$G\left(L^{\prime },\;L\left(t\right)\right)=\frac{1}{\sqrt{2\pi \sigma_{L}\left(L\left(t+1\right)\right)}}e^{\frac{-(L^{\prime }-E{\left(L\left(t+1\right)\right)}^{2}}{2\sigma_{L}^{2}\left(L\left(t+1\right)\right)}}$$

with

(4) $$E\left(L\left(t+1\right)\right)=L\left(t\right)e^{-{\dot{r}}_{B}}+\left(1-e^{-{\dot{r}}_{B}}\right)L_{m}\cdot E\left(Y\right),$$

and

(5) $$\sigma^{2}\left(L\left(t+1\right)\right)={\left(1\;-\;e^{-{\dot{r}}_{B}}\right)}^{2}L_{m}^{2}\sigma^{2}\left(Y\right),$$

where ${\dot{r}}_{B}$ is known as the von Bertalanffy growth rate and σ(*Y*) is the standard deviation of the expected feeding level. Note that Equations 4 and 5 assume that individuals can shrink under starvation conditions.

The reproduction function $R\left(L\left(t\right)\right)$ gives the number of offspring produced between time *t* and *t* + 1 by an individual of length *L* at time *t*:

(6) $$R\left(L(t)\right)=\left\{\begin{array}{cc} 0 & \;\text{for}\;\;L_{b}\leq L<L_{p}\\ E\left(Y\right)\cdot R_{m}L{\left(t\right)}^{2}/L_{m}^{2} & \;\text{for}\;\;L_{p}\leq L\leq L_{m}E\left(Y\right)/\kappa \end{array}\right.$$

where *R*_*m*_ is the rate at which female offspring are produced by an adult female of maximum length *L*_*m*_. Individuals are mature when they reach maturity (“puberty”) at length *L*_*p*_ and only surviving adults reproduce; therefore, only individuals within a cohort of length *L*_*p*_ ≤ *L* ≤ *L*_*m*_ *Y*/κ reproduce.

The probability density function $D\left(L^{\prime },\;L\left(t\right)\right)$ gives the probability that the offspring of an individual of length *L* are of length *L*′ at time *t* + 1, and hence describes the association between parent and offspring character values:

(7) $$D\left(L^{\prime },L\left(t\right)\right)=\left\{\begin{array}{cc} 0 & \mathrm{for}\;L<L_{p}\\ \frac{1}{\sqrt{2\pi \sigma_{L_{b}}\left(L\left(\;t\right)\right)}}e^{\frac{-(L^{\prime }-E_{L_{b}}{\left(L\left(t\right)\right)}^{2}}{2\sigma_{L_{b}}^{2}\left(L\left(\;t\right)\right)}} & \mathrm{otherwise} \end{array}\right.\;$$

where $E_{L_{b}}\left(L\left(t\right)\right)$ is the expected size of offspring produced by a cohort of individuals with length *L*(*t*), and $\sigma_{L_{b}}^{2}\left(L\left(t\right)\right)$ is the associated variance.

### 2.4 DEB‐IPM parameterisation

We parameterised the DEB‐IPM using the same values as previously described (Smallegange et al., 2017) (Table 1): we set *L*_*b*_ = 0.166 mm, *L*_*m*_ = 1.008 mm, κ = 0.083 and μ = 0.03 d^−1^. Bulb mites are very plastic in their size at maturity in response to food conditions, so, as before, we related *L*_*p*_ to *L*_∞_ using the linear relationship *L*_*p*_ = 0.539 · *L*_∞_, where the ultimate length *L*_∞_ is the asymptote of the von Bertalanffy growth curve in length and represents the largest length an individual can achieve at a particular feeding level. Ultimate length can be calculated from the expected feeding level and maximum length as $L_{\infty }=L_{m}\cdot E\left(Y\right)$ (Kooijman & Metz, 1984). Plasticity in the timing of maturation emerges from the growth process, which can be slow under low feeding levels (low environmental quality) and high under high feeding levels (high environmental quality) (Equation 4). Because mites also exhibit very plastic growth in response to different feeding levels (i.e. environments of different quality), ${\dot{r}}_{B}$ is related to feeding level and ultimate length following ${\dot{r}}_{B}=1/\left(151.0-137.8\cdot L_{\infty }\right)$. Finally, as egg length at birth is independent of feeding level and maternal length (Smallegange et al., 2014), we set $E_{L_{b}}\left(L\left(t\right)\right)=L_{b}=0.166$ mm and $\;\sigma_{L_{b}}^{2}\left(L\left(t\right)\right)\;=0$. We did not use data from our population experiment, as, in contrast to the population experiment, these life‐history data were scored under standardised, *ad libitum* food conditions, in line with the assumptions of the DEB growth model (Kooijman & Metz, 1984).

**Table 1:** Dynamic energy budget parameters for female bulb mites (Smallegange et al., 2017)

Symbol	Description	Value	Unit
*E*(*Y*)	Expected feeding level	—	—
*L*_*b*_	Body length at birth	0.166	mm
*L*_*p*_	Body length at maturity (“puberty”)	$0.539\cdot L_{\infty }$	mm
*L*_∞_	Ultimate length	$L_{m}\cdot E\left(Y\right)$	mm
*L*_*m*_	Maximum length at *E*(*Y*) = 1	1.008	mm
*R*_*m*_	Maximum rate of producing female offspring at *L*_*m*_	16	# d^−1^
${\dot{r}}_{B}$	von Bertalanffy growth rate	$1/\;\left(151.0-137.8L_{\infty }\right)$	d^−1^
κ	Energy allocation fraction to somatic maintenance and growth	0.083	—
μ	Mortality rate	0.03	d^−1^
σ(*Y*)	Standard deviation of *E*(*Y*)	0.3	—

### 2.5 Cross‐level test: simulating the experiment using the DEB‐IPM

To simulate the experiment, we used the population model $p\left(t+1\right)=A\left(t\right)p\left(t\right)$, where **p**(*t*) is the population vector at time *t* (only females) and **A**(*t*) is the DEB‐IPM at time *t* defined by the feeding level *E*(*Y*) at time *t*. We created two sequences of 31 days (acclimation) + 148 days (experiment). During the acclimation period, mites were fed five yeast rods per day. The experimental period comprised a sequence of low and high *E*(*Y*) values, representing two and eight yeast rods, respectively, that the populations received each day under either the blue, white or red noise treatments. To construct the matrix **A**(*t*), we discretised the IPM (Equation 1) and divided the length domain Ω into 200 very‐small‐width discrete bins, defined as “mesh points” (a higher number of bins did not produce different results). Then, at each step in a sequence of feeding levels, the value of *E*(*Y*), in addition to the parameter values given for bulb mites in Table 1, served as input for the individual‐level functions to create a matrix **A**(*t*) that mapped **p**(*t*) of 200 size classes from time *t* to *t *+ 1. As in the experiment, we started with 20 adults. Within each noise colour, we simulated the harvesting treatment of the experiment by, every eighth day, removing 50% of adults that were larger than the actual observed threshold length for harvesting. This threshold length was calculated by taking the ratio of the mean length of harvested adult females to the mean length of all adult females (which equalled 1.0235: Appendix S1 in Supporting Information). At the end of each noise treatment simulation, we averaged the log‐transformed numbers of eggs, juveniles and adults over the experimental period to compare them with the observed counts. We also calculated the log‐stochastic population growth rate λ_s_ over this period τ, as before. Because we did not know the exact feeding levels experienced by individuals in the experimental populations, we calculated the latter response variables for all pairwise combinations of low and high feeding levels where $E{\left(Y\right)}_{\mathrm{low}}$ was within the range $0.10\;\leq \;E{\left(Y\right)}_{\mathrm{low}}<0.36$ and $E{\left(Y\right)}_{\mathrm{high}}$ was within the range $0.36\;\leq \;E{\left(Y\right)}_{\mathrm{high}}\leq 0.60$. These values were chosen because our previous work revealed that bulb mite populations are constant and stable at a feeding level of *E*(*Y*) = 0.36 (Figure S5 in Smallegange et al., 2017). Therefore, $E{\left(Y\right)}_{\mathrm{high}}$ values always elicit population growth, whereas $E{\left(Y\right)}_{\mathrm{low}}$ values elicit population decline. For the cross‐level test, we investigated to what extent the 95% confidence intervals of our observed mean log‐transformed numbers of females in each life stage and population growth rate overlapped with the predicted values of mean log‐transformed life stage numbers and λ_s_.

### 2.6 Stochastic demographic model

A stochastic demographic model was constructed using a two‐state Markov chain that gave the probability distribution of environment states at time *t*. In this chain, state 1 was the good environment and state 2 was the bad environment, which resulted in the following Markov chain transition matrix **M** (Caswell, 2001, p. 379):

(8) $$M=\left[\begin{array}{cc} 1-p & q\\ p & 1-q \end{array}\right]$$

where *p* is the probability of switching from the good to the bad environment and *q* is the probability of switching from the bad to the good environment. The serial or autocorrelation of the Markov chain equals ρ = 1 − *p* − *q* (Caswell, 2001; p. 379). High, positive values of ρ denote red noise; high, negative values of ρ denote blue noise; and ρ = 0 denotes white noise, in which the probability of switching states is independent of the current state. The temporal frequency at which the good environment occurs is given by *f* = *q*/(*p* + *q*) (Caswell, 2001, p. 379). We used a combination of the highest and lowest feeding levels at which we observed population equilibrium at log(λ_s_) = 0 in the cross‐level test to define the good and bad environment states. This resulted in an expected feeding level of $E{\left(Y\right)}_{\mathrm{high}}=0.60$ to represent the good environment and $E{\left(Y\right)}_{\mathrm{low}}=0.15$ to represent the bad environment (see Section 3).

By iterating **M** for all combinations of *p* and *q* for each combination of $E{\left(Y\right)}_{\mathrm{low}}$ and $E{\left(Y\right)}_{\mathrm{high}}$, a time series of length *S* = 3,000 (with an initial transient length of 500 discarded, a starting population of one individual in each size bin, and with the initial environment state chosen randomly (see also Tuljapurkar et al., 2003)) was generated (e.g. Smallegange et al., 2014). As before, this sequence determined the environment state, and hence the feeding level *E*(*Y*) that a population experienced at each time‐step, from which the individual‐level functions were calculated to construct **A**(*t*). At each time‐step, **A**(*t*) was stored with associated vectors of population structure to calculate the stochastic population growth rate λ_s_, as above, where τ = 3,000 – 500 = 2,500. We then repeated this exercise by imposing the same harvesting regime as in the experiment: every 8th day, 50% of adults that were larger than the ratio of the mean length of harvested adult females to the mean length of all adult females (1.0235; Appendix S1 in Supporting Information) were harvested. For both unharvested and harvested scenarios, we examined the elasticity of λ_s_ to perturbation of each of the model parameters *L*_*b*_, *L*_*p*_, *L*_*m*_, *R*_*m*_, ${\dot{r}}_{B}$, κ and μ (Table 1) to identify which life‐history traits under which stochastic and/or harvesting regimes were most influential to the long‐run stochastic population growth rate λ_s_. For this, we perturbed each parameter by 1% and calculated the elasticity of λ_s_ to each model parameter.

## 3 RESULTS

### 3.1 Experiment

**Figure 1:**
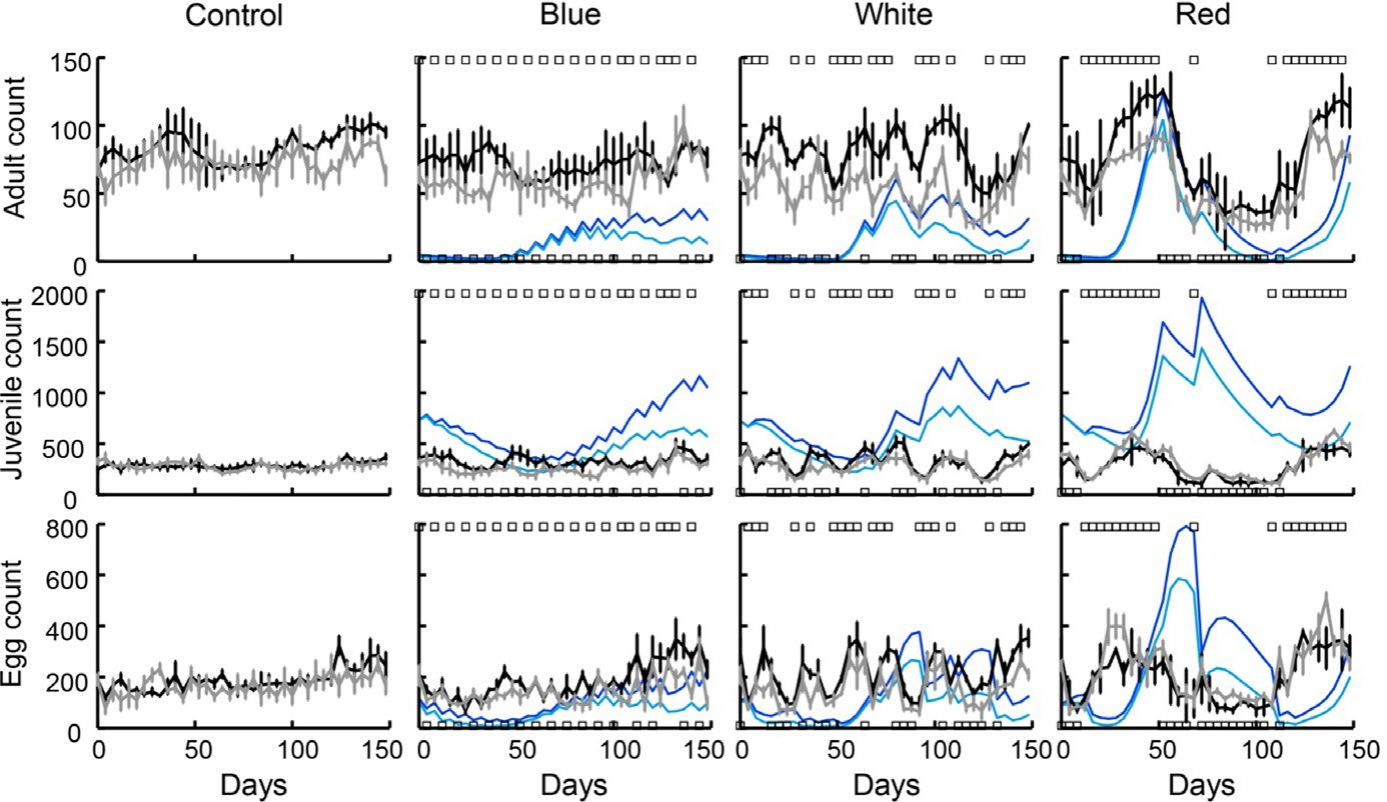
Observed (black and grey lines) and predicted (blue lines) time series for unharvested populations (black and dark blue) and large‐adult harvested populations (grey and light blue) in constant and temporally variable food environments. The observations (black and grey lines) are means ± SE for all replicates for constant food availability (control), and temporal variation in food availability that has a blue, white or red colour. Rows are (top) adult numbers (both males and females), (middle) juvenile numbers and (bottom) egg numbers. In each panel, a predicted line is a single simulation run of the experiment (see: Cross‐level test in the Methods) for either unharvested (dark blue) or harvested (light blue) populations, where $E{\left(Y\right)}_{\mathrm{low}}=0.25$ and $E{\left(Y\right)}_{\mathrm{high}}=0.45$. Square symbols in the stochastic food environment panels denote whether populations were provided with eight yeast rods (upper squares) or two yeast rods (lower squares) each day since the last census moment. In the constant food treatment, populations were fed five yeast rods every day. Time zero is preceded by the acclimation period of 31–34 days during which time populations were fed five yeast rods per day

Under the constant food regime, both unharvested and harvested bulb mite populations show stable dynamics (Figure 1: black and grey lines). The dynamics of populations in environments where temporal variation in food availability has a white or red colour are markedly different from the inherently equilibrium dynamics of the populations under constant food conditions (Figure 1). Regardless of whether harvesting occurs, all life stages track food availability under both white and red environmental variation: counts are low (or high) when food availability is low (or high) (Figure 1). This pattern is less clear under blue environmental variation (when fluctuations in food availability occur at high frequency) where fluctuations in both harvested and unharvested populations appear similar to, but more variable than those observed under constant food conditions (Figure 1).

**Figure 2:**
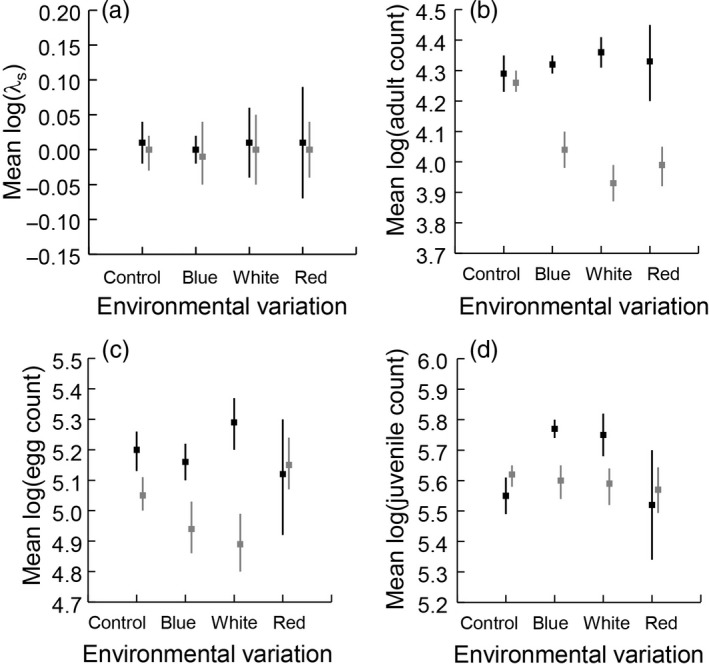
Mean log‐transformed stochastic population growth rate λ_s_ (a), adults (both males and females) (b), eggs (c) and juveniles (d) graphed by environmental variation treatment group (control [constant food], blue, white and red) for unharvested populations (black) and large‐adult‐harvested populations (grey). Vertical lines are bias‐corrected, and adjusted (BCa) 95% confidence intervals (CIs) around the observed means were estimated by bootstrap resampling (by taking 1000 resamples, stratified by population tube) using the *boot* package in r

We first focused on the overall population response, mean log(λ_s_). Mean log(λ_s_) was always slightly, non‐significantly lower for the harvested than the unharvested populations (*t* = −0.22, *p* = .414), and was unaffected by the type of environmental variation that the populations experienced (*t* = 0.32, *p* = .127) or their interaction (*t* = −0.15, *p* = .441) (Figure 2a). Next, we examined the life stage counts. For adults, the interaction between harvesting and environmental variation was significant (interaction: *t* = −4.17, *p* = .001), because harvesting significantly reduced mean adult numbers under each of the environmental variation treatments, but only marginally under the control treatment (Figure 2b). The effect of adult harvesting carried over to reduce egg numbers under the blue, white and control environmental variation treatments, but not under the red environmental variation treatment (interaction: *t* = 4.30, *p* < .001) (Figure 2c). The overall effect of environmental variation on egg numbers was non‐significant (*t* = 1.25, *p* = .110), whereas the overall effect of harvesting significantly reduced the mean number of eggs (*t* = −4.83, *p* < .001) (Figure 2c). Regarding the juveniles that developed from the eggs, the effect of adult harvesting carried over to significantly reduce mean juvenile numbers under the blue and white environmental variation treatments, but not under the red or control treatments (significant interaction between harvesting and environmental variation: *t* = 5.94, *p* < .001) (Figure 2d).

### 3.2 Cross‐level test

**Figure 3:**
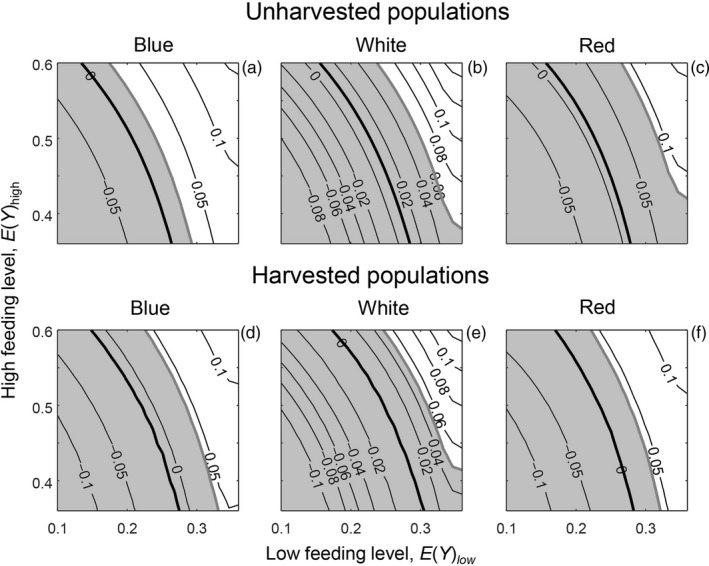
Contour plot where thin black lines show predicted log(λ_s_) in relation to the low feeding level *E*(*Y*)_low_ (representing two yeast rods of food a day) and the high feeding level *E*(*Y*)_high_ (representing eight yeast rods of food a day) for unharvested populations (a–c) and populations in which the largest adults were harvested (d–f) in relation to environmental variation treatments in which temporal variation in food availability had a blue, white or red colour. The thick black lines are predicted means and the shaded areas bordered by thick grey lines indicate the 95% confidence intervals around the predicted mean. Observed values (thin lines) that fall within the shaded area are not significantly different from the model predictions

**Figure 4:**
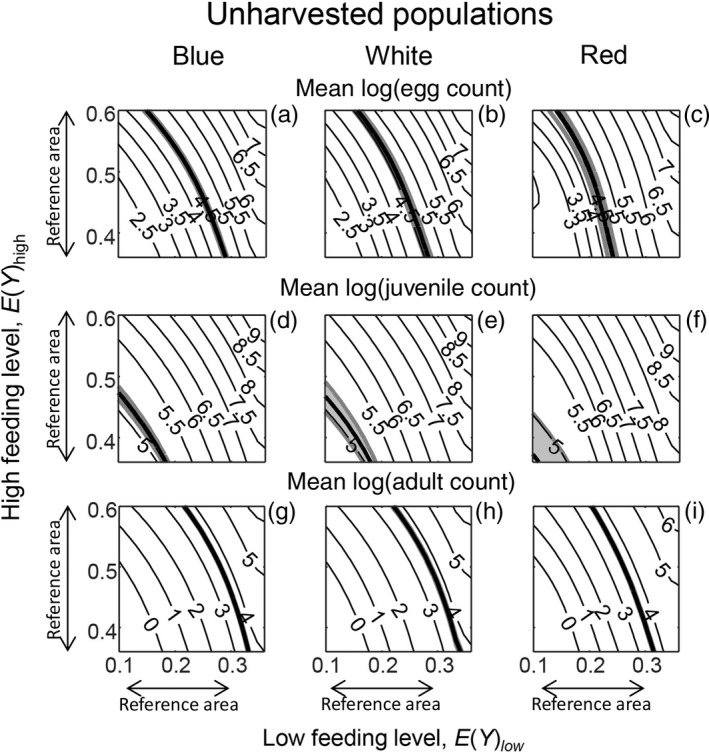
Contour plot where thin black lines show, for unharvested populations, predicted mean log‐transformed life stage counts in relation to the low feeding level *E*(*Y*)_low_ (representing two yeast rods of food a day) and the high feeding level *E*(*Y*)_high_ (representing eight yeast rods of food a day) in relation to environmental variation treatments in which temporal variation in food availability had a blue, white or red colour. Life stages concern female eggs (a–c), juveniles (d–f) and adults (g–i). The thick black lines are predicted means and the shaded areas bordered by thick grey lines indicate the 95% confidence intervals around the predicted mean. Observed values (thin lines) that fall within the shaded area are not significantly different from the model predictions. Also indicated along the x‐ and y‐axes is the cross‐level test reference area: the ranges of low and high feeding levels in which population equilibria occurred ($0.13\leq E{\left(Y\right)}_{\text{low}}<0.30$, and $0.36\leq E{\left(Y\right)}_{\text{high}}<0.60$ [Figure 3])

**Figure 5:**
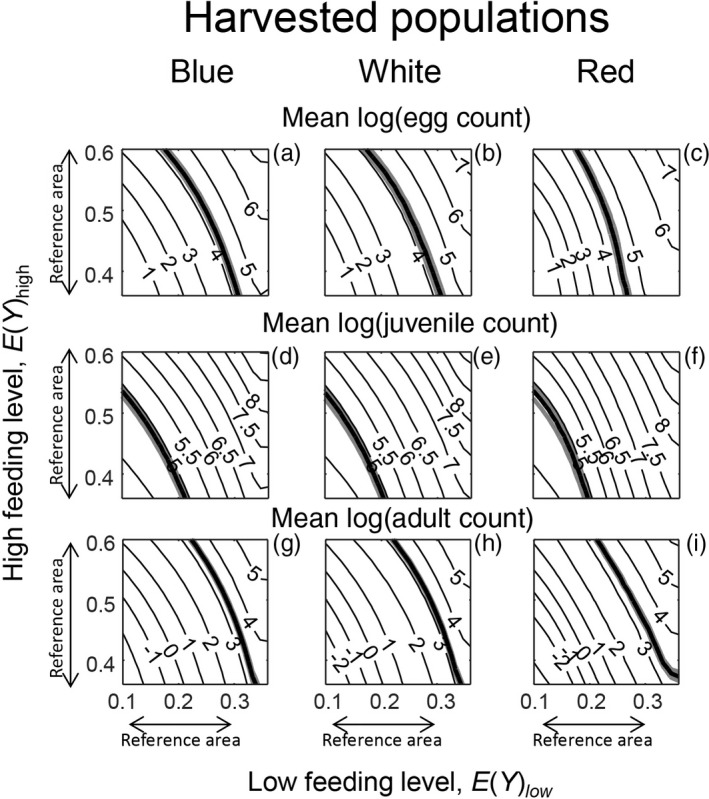
Contour plot like Figure 4, but for harvested populations. See further Figure 4

We first examined the overall population response, i.e. the population growth rate. The areas in which the bulb mite populations were predicted to be at equilibrium, i.e. where log(λ_s_) = 0, were well within the range of low and high feeding levels investigated (Figure 3: thin black lines are predictions; the observed mean log(λ_s_) value for each environmental variation treatment is indicated by a thick black line). These areas of population equilibrium occurred across very similar ranges of low and high feeding levels across the three environmental variation treatments (Figure 3), as in the experiment (Figure 2a). In addition, as in the experiment, predicted log(λ_s_) was always slightly lower for any combination of low and high feeding levels for the harvested populations than the unharvested ones. These observations suggest that the model performed well at the population level. For the cross‐level tests at the life stage level, the ranges of low and high feeding levels in which population equilibria occurred are henceforth referred to as the cross‐level test reference area: $0.13\leq E{\left(Y\right)}_{\mathrm{low}}<0.30$, and $0.36\leq E{\left(Y\right)}_{\mathrm{high}}<0.60$ (Figure 3). The life stage counts of the unharvested populations revealed that the overlap between the predicted and observed adult and egg counts mainly occurred within the cross‐level test reference area (Figure 4: top and bottom rows: the reference area is denoted by arrows). In contrast, the predicted juvenile counts within this reference area were much higher than the observed juvenile counts, which were mainly outside the reference area (Figure 4: middle row: the reference area is denoted by arrows). For the harvested populations, the model predictions of juvenile counts were lower, but were still mostly overestimated within the reference area (Figure 5: middle row: the reference area is denoted by arrows). The predictions of adult and egg counts in the harvested populations mainly overlapped with the observed counts within our reference area, as in the unharvested populations (Figure 5: top and bottom rows: the reference area is denoted by arrows). As a visual aid of our findings that predicted and observed adult and egg counts were comparable, but predicted and observed juvenile counts were not, we ran single simulations for all experimental treatments where we (fairly arbitrarily) set $E{\left(Y\right)}_{\mathrm{low}}=0.25$ and $E{\left(Y\right)}_{\mathrm{high}}=0.45$. This showed that, for both harvested and unharvested populations, predicted juvenile counts were mostly overestimated, whereas predicted adult and egg counts were more comparable to observed counts (Figure 1: blue lines are the simulations).

### 3.3 Stochastic demographic model

**Figure 6:**
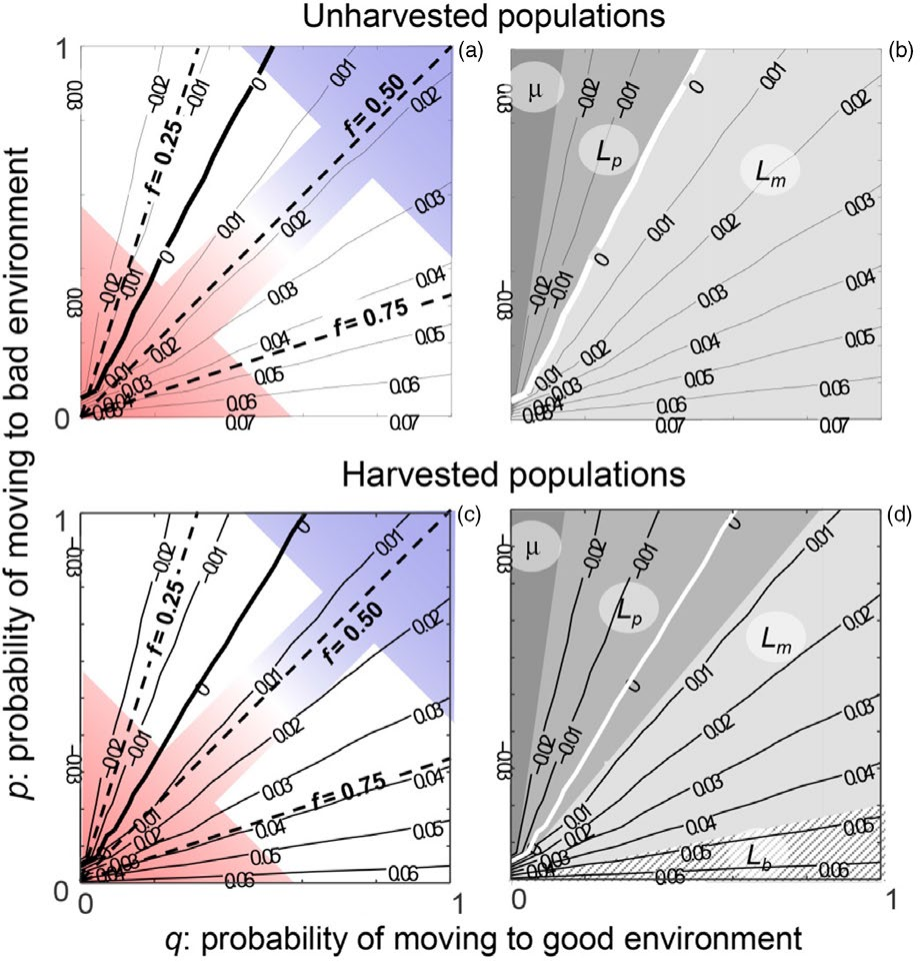
Contour plots showing how the log‐stochastic population growth rate, log(λ_s_), relates to the probability of switching from the bad to the good environment, *q*, and the probability of switching from the good to the bad environment, *p*, for both unharvested (a) and harvested (b) populations. The coloured arrows in (a) and (c) show values of *p* and *q* where the autocorrelation, ρ, in environmental regimes was red (bottom‐left corner), white (antidiagonal) and blue (top‐right corner); the three dashed lines in (a) and (c) show values of *p* and *q* where the good environment frequency *f* equalled 0.25, 0.50 and 0.75. In each plot, the thick solid line denotes log(λ_s_) = 0. The different grey areas in (b) and (d) denote for which combination of *p* and *q*, which life‐history parameter (dark grey: morality rate μ; intermediate grey: length at maturity *L*_*p*_; light grey: maximum length *L*_*m*_; dashed area: length at birth *L*_*b*_) was λ_s_ most sensitive to. In all panels, $E{\left(Y\right)}_{\mathrm{low}}=0.15$ and $E{\left(Y\right)}_{\mathrm{high}}=0.60$

Analysis of the stochastic demographic model revealed that for both the unharvested and harvested populations, increases in log(λ_s_) closely matched increases in the frequency of good environments, *f* (Figure 6a,c: solid lines denoting constant values of log(λ_s_) are parallel to dashed lines denoting constant values of *f*). Therefore, at intermediate values around *f* = 0.5, as in the experiment, shifts in noise colour (i.e. the autocorrelation, ρ) had no relationship with variation in log(λ_s_) (Figure 6a: solid lines denoting constant values of log(λ_s_) close to the diagonal where *f* = 0.5 are almost perpendicular, and hence unrelated, to constant values of ρ, denoted by the coloured arrow). In contrast, at low values of *f*, log(λ_s_) increased as noise colour shifted from red to blue (moving parallel to the diagonal in the top‐left corners of Figure 6a). In contrast, at high values of *f*, log(λ_s_) decreased as noise colour shifted from red to blue (moving parallel to the diagonal in the bottom‐right corners of Figure 6a). As before, the effect of selectively harvesting large adults on log(λ_s_) was small, because for each combination of $E{\left(Y\right)}_{\mathrm{low}}$ and $E{\left(Y\right)}_{\mathrm{high}}$, log(λ_s_) was slightly lower for the harvested than the unharvested populations (Figure 6a [unharvested populations] vs. Figure 6c [harvested populations]). A perturbation analysis of the unharvested populations revealed that log(λ_s_) was most sensitive to perturbation of *L*_*m*_ for log(λ_s)_ > 0 (Figure 6b: light grey area), but for log(λ_s_) < 0, log(λ_s_) was most sensitive to perturbation of *L*_*p*_ (Figure 6b: intermediate grey area), and for the lowest values of log(λ_s_), most sensitive to perturbation of μ (Figure 6b: dark grey area). For the harvested populations, the shift from *L*_*p*_ to *L*_*m*_ being the most influential parameter to λ_s_ occurred at a much higher, positive value of log(λ_s_) (Figure 6d). At the highest values of the good environment frequency *f*, λ_s_ was most sensitive to perturbation of *L*_*b*_ (Figure 6d), in contrast to in the unharvested populations, in which *L*_*m*_ was the most influential (Figure 6b).

## 4 DISCUSSION

Here we tested to what extent a DEB‐IPM population model in which model parameters represent individual life‐history traits (as opposed to statistical coefficients in standard IPMs) accurately predicts population responses to perturbation of bulb mite adult survival in stochastic environments. Our experimental results revealed that across all environmental variation treatments, harvesting slightly reduced the log of the stochastic population growth rate, or average fitness (although for other organisms, the harvesting rates we applied could have much greater effects, Sæther et al., 2005). Predictions from the DEB‐IPM matched this overall population response, from which we infer that our cross‐level test on overall population performance was satisfactory. The model therefore likely captures the overall effect of density dependence through food exploitation very well, as population equilibrium is reached across the combinations of expected feeding levels of which we know are close to levels for which we observe population equilibrium in our experimental populations (Smallegange et al., 2017: Appendix S1). In addition, the more general analysis of population performance across a wide range of stochastic environments showed that the pattern of a small reduction in average fitness under large‐adult harvesting persisted across different noise colours and frequencies of good environments. However, when examining the responses of the different life stages, a different pattern emerged. In our experiment, similar to findings by Cameron et al. (2016) for *S. berlesei*, the effect of harvesting on life stage counts depended on the colour of the environmental noise. In all environments, large‐adult harvesting reduced the mean adult counts; however, in red environments, in contrast to the blue or white environments, this reduction in adult counts did not carry over to reduce egg and juvenile counts. It is possible that during the relatively long periods of high food conditions under red noise, which occurred for about half of the experimental period (Appendix S1 in Supporting Information), adults could increase their reproductive output, and juveniles increase their maturation rates, thereby compensating for the increased adult mortality. The DEB‐IPM, however, did not predict this compensatory response in egg production and subsequent compensation in juvenile counts under red noise. Moreover, across the environmental variation treatments, like previous cross‐level tests (Martin, Jager, Nisbet, Preuss, & Grimm, 2013; van der Meer, 2016), we found that the DEB‐IPM overestimated juvenile counts, although this mismatch was slightly reduced under large‐adult harvesting. There could be several explanations for this. For example, we know that mite life histories can be very plastic in response to density and maternal effects (Benton, Clair, & Plaistow, 2008; Benton, Plaistow, Beckerman, Lapsley, & Littlejohns, 2005; Cameron & Benton, 2004), which affects mite population dynamics (Beckerman, Benton, Lapsley, & Koesters, 2003). Such effects are not yet included in the DEB‐IPM. van der Meer (2016), in turn, states that, in DEB theory, juveniles can survive unfavourable (starvation) conditions better than adults due to the way that energy uptake and somatic maintenance are modelled, creating a mismatch between predicted and observed population structures. We therefore suggest future work to focus on including into the DEB core: maternal effects, delayed effects of plasticity, interference competition within and between life stages (Cameron, Wearing, Rohani, & Sait, 2007), and how these are affected by environmental variability (cf. Beckerman et al., 2003). This may solve the fundamental problem of why DEB models that accurately describe individual‐level life‐history patterns fail to accurately predict population dynamics (van der Meer, 2016).

When a model, like the DEB‐IPM, is built from parameters that represent individual traits, it is possible to study the functional links between individual life history, the environment and population dynamics. We will illustrate this using two examples. Firstly, our stochastic perturbation analysis revealed that the life‐history parameter that affected the stochastic population growth rate the most across most stochastic environments was “maximum length” (*L*_*m*_, see Table 1). We can therefore ask the question whether there is (empirical) evidence that maximum body length indeed influences population growth. In the DEB‐IPM, maximum length, together with feeding level, determines ultimate adult length (Table 1). Smallegange and Deere (2014) observed in their long‐term population experiment on bulb mites that not only the mean ultimate length (*L*_∞_, see Table 1) of adult female bulb mites increased over about 15 generations but egg production, and consequently population size, also increased (Smallegange & Deere, 2014). Does this mean that there is a direct link between maximum length and population growth? Maximum length is positively related to maximum ingestion rate (Kooijman & Metz, 1984), and, as feeding levels were almost constant throughout Smallegange and Deere’s (2014) experiment, an evolutionary or plastic increase in maximum ingestion rate, resulting in an increase in maximum length, could explain the observed increase in ultimate length. Crucially, maximum ingestion rate is positively related to maximum reproduction rate (Kooijman & Metz, 1984), and an increase in maximum ingestion rate, together with the fact that larger adult females lay more eggs (Smallegange, 2011), could explain the concurrent increases in egg number and total population size (Smallegange & Deere, 2014). Our second example concerns the observation that λ_s_ was highly and positively correlated with the frequency with which periods of good environments (with high feeding levels) occurred. Given the fact that the good environment frequency and the autocorrelation, or noise colour, are functionally related through their dependency on the probabilities of switch environmental states, *p* and *q* (see Methods), λ_s_ is predicted to increase when moving from red to blue noise at low good environment frequencies like *f* = 0.25, but the opposite is predicted to occur at high good environment frequencies like *f* = 0.75. It is unclear whether this result is specific to bulb mites, or perhaps generalisable to fast, plastic life‐history organisms, like bulb mites. This is important to find out, particularly for the management of threatened species, because a pattern of decreasing population performance with shifts from red to blue environmental noise colour can have ramifications for population persistence, as climate variables, at least on a continental scale, are shifting from a more red to a more blue colour (García‐Carreras & Reuman, 2011).

Do our experimental results also have any repercussions for harvesting theory? Single‐species models have been used extensively to study population responses to harvesting (Ruokolainen, Lindén, Kaitala, & Fowler, 2009). One general pattern that has emerged is how noise colour interacts with density dependence (Kaitala et al., 1997): a population with undercompensating growth reacts slowly to environmental changes, whereas overcompensating populations overshoot their equilibrium after a perturbation. Bulb mite population growth has been characterised as overcompensating (Cameron & Benton, 2004; Cameron et al., 2016). We found that the type of compensating response in the mite populations to the single harvesting rate that we investigated was an interaction between noise colour and harvesting rate, as compensation to harvesting in terms of population growth was not achieved, thus showing undercompensation. The type of compensating responses of the different life stages to selective harvesting, in turn, depended on noise colour. Indeed, Wikström, Ripa, and Jonzén (2012) predicted from their age‐structured model with stochastic recruitment that proportional adult harvesting leads to stabilising effects only in a small percentage of cases. These potentially complex responses to environmental stochasticity highlight the need to unravel the underlying mechanisms of how environmental variables translate into population‐dynamical processes via individual life‐history traits and competition, not just at the level of species (e.g. Sæther et al., 2005), but at different scales of organisation (Ruokolainen et al., 2009). This is particularly pertinent now as long‐term climate change is causing ever greater environmental fluctuations (Wigley, Smith, & Santer, 1998).

In conclusion, understanding population dynamics is ultimately a question of understanding the interaction between the environment and individual life histories. We have given some examples of the types of questions on these interactions that can be explored when parameters of mechanistic IPMs, like the DEB‐IPM, represent individual life‐history traits. Such a mechanistic IPM also opens up other possibilities. For example, tools like bifurcation analysis can now be used to study model dynamics as a function of the values of these life‐history traits (e.g. Smallegange et al., 2017: Appendix S1) so that one could e.g. ask “at what maximum length or mortality rate do abrupt changes, like from population stability to extinction, occur?” Such analyses could be particularly insightful when investigating the intricacies of life‐history effects on population dynamics. For example, soil mite dynamics are affected by differential egg provisioning (Benton et al., 2005), maternal‐age effects (Benton et al., 2008) and delayed effects of plasticity in density‐dependent growth and reproduction (Beckerman et al., 2003). To some extent, these aspects could be explored using bifurcation analyses of ${\dot{r}}_{B}$ or *R*_*m*_, but more detailed DEB growth models are required to explore age and delayed effects. Finally, one could use quantitative genetics to create an evolutionary explicit DEB‐IPM (cf. Coulson et al., 2017) to study concurrent ecological and evolutionary dynamics. Because the parameters of the DEB‐IPM represent life‐history traits, this is straightforward as one could apply the animal model (Kruuk, Slate, & Wilson, 2008) to observational data on the life‐history traits of related individuals to divide each model parameter into its heritable genetic and environmental component. First and foremost, however, a general solution should be found for the “DEB in population models paradox” (van der Meer, 2016).

## ACKNOWLEDGEMENTS

We thank Editor Dylan Childs for constructive comments on an earlier version of the manuscript. I.M.S. acknowledges funding from the Netherlands Organisation for Scientific Research (NWO) (MEERVOUD grant 836.13.001 and VIDI grant 864.13.005) and an ERC Advanced Grant no. 249872 awarded to Tim Coulson. H.M.E. was supported by the Volkert van der Willigen Fonds of the Stichting Amsterdamse UniversiteitsFonds.

## AUTHORS’ CONTRIBUTIONS

I.M.S. and H.M.E. conceived the ideas and designed methodology. H.M.E. collected the data. I.M.S. analysed the data and led the writing of the manuscript. H.M.E. contributed critically to the drafts and gave final approval for publication.

## DATA ACCESSIBILITY

Data, R code and MatLab code are available from figshare: https://doi.org/10.6084/m9.figshare.5356051 (Smallegange, 2018).
