# Supplementary material for: Trait‐based predictions and responses from laboratory mite populations to harvesting in stochastic environments
Source: J Anim Ecol. 2018 Jun 17;87(4):893–905. doi: 10.1111/1365-2656.12802 (PMC6032940; doi:10.1111/1365-2656.12802)
Supplement: Supplementary file 1 [file JANE-87-893-s001.docx]

**Appendix S1. Supporting Information to:
Trait-based predictions and responses from laboratory mite
populations to harvesting in stochastic environments**

Isabel M. Smallegange^*^ and Hedwig M. Ens

Institute for Biodiversity and Ecosystem Dynamics (IBED), University of Amsterdam,

P.O. Box 94240, 1090 GE Amsterdam, The Netherlands

^*^ i.smallegange@uva.nl

**Contents**:

Assessing the threshold harvesting length

**Assessing the threshold harvesting length**

In the experiment, the harvesting of the largest adults was done by eye (see *Methods* in the main text). To simulate this selective harvesting, we needed to know the threshold body length for selecting large adult females that was used in the experiment. To this end we measured the length of adult females on four separate occasions: 8, 16, 24 and 32 days after starting the experiment. On each of these days, we measured all harvested adult females (ranging between 12 and 29 females) and between 5 and 10 adult females randomly selected from unharvested populations. We photographed each female using an Axiocam 105 digital colour camera connected to a Zeiss Stemi 2000C (6.5–50x) stereomicroscope, and measured their longest body length (idiosoma length) to the nearest 0.1 μm using ZEN lite imaging software (Zeiss). We next used a generalised linear mixed model (GLMM) with Gaussian errors (using the *lme4* package in R [R Development Core Team, 2013]) to assess the effect of the fixed factors harvesting treatment (no harvesting, harvesting) and environmental variation treatment (control, blue, white and red noise), with “measurement day” and “population tube” as random effects to account for the repeated measures within each experimental population, on female body length. The model assumptions of Gaussian errors and homoscedacity were confirmed by inspecting the probability plots and error structures. One population tube was dropped during a census and excluded from the analyses.

We found that harvested females were significantly larger at 546.25 ± 0.07 SE mm than randomly selected females from unharvested populations, which were on average 533.71 ± 0.22 mm (*t* = 2.28, p = 0.012). The overall ratio of mean body length of harvested females over non-harvested females therefore equalled 546.25 μm / 533.71 μm =1.0235; which we used in the simulations. There was no significant effect of the type of environmental variation that the populations experienced (*t* = 0.63, p = 0.265), or the interaction between environmental variation and harvesting (*t* = 0.29, p = 0.386).
